# Modifying Choroidal Neovascularization Development with a Nutritional Supplement in Mice

**DOI:** 10.3390/nu7075229

**Published:** 2015-07-06

**Authors:** Alina Adriana Ivanescu, Patricia Fernández-Robredo, Henar Heras-Mulero, Luis Manuel Sádaba-Echarri, Laura García-García, Vanessa Fernández-García, Maite Moreno-Orduna, Aitor Redondo-Exposito, Sergio Recalde, Alfredo García-Layana

**Affiliations:** 1 Experimental Ophthalmology Laboratory, School of Medicine, University of Navarra, Irunlarrea s/n Street, Los Castaños Building, 31008 Pamplona, Spain; E-Mails: alina_adriana83@hotmail.com (A.A.I.); pfrobredo@unav.es (P.F.-R.); mgarcia.6@alumni.unav.es (L.G.-G.); vfernandez87@hotmail.es (V.F.-G.); maimoreno@unav.es (M.M.-O.); aredondo.5@alumni.unav.es (A.R.-E.); srecalde@unav.es (S.R.); 2 Department of Ophthalmology, Hospital Virgen del Camino, Servicio Navarro de Salud, Irunlarrea 4 Street, 31008 Pamplona, Spain; E-Mail: henarheras@yahoo.es; 3 IdiSNA, Navarra Institute for Health Research, 31008 Pamplona, Spain; 4 Department of Ophthalmology Clínica Universidad de Navarra, University of Navarra, Avda Pio XII, 36, 31008 Pamplona, Spain; E-Mail: lmsadaba@unav.es

**Keywords:** antioxidants, choroidal neovascularisation, mouse model, anti-VEGF

## Abstract

We examined the effect of nutritional supplements (modified Age Related Eye Disease Study (AREDS)-II formulation containing vitamins, minerals, lutein, resveratrol, and omega-3 fatty acids) on choroidal neovascularization (CNV). Supplements were administered alone and combined with intravitreal anti-VEGF in an early-CNV (diode laser-induced) murine model. Sixty mice were evenly divided into group V (oral vehicle, intravitreal saline), group S (oral supplement, intravitreal saline), group V + aVEGF (oral vehicle, intravitreal anti-VEGF), and group S + aVEGF (oral supplement, intravitreal anti-VEGF). Vehicle and nutritional supplements were administered daily for 38 days beginning 10 days before laser. Intravitreal injections were administered 48 h after laser. Fluorescein angiography (FA) and flat-mount CD31 staining evaluated leakage and CNV lesion area. Expression of VEGF, MMP-2 and MMP-9 activity, and NLRP3 were evaluated with RT-PCR, zymography, and western-blot. Leakage, CNV size, VEGF gene and protein expression were lower in groups V + aVEGF, S + aVEGF, and S than in V (all *p <* 0.05). Additionally, MMP-9 gene expression differed between groups S + aVEGF and V (*p <* 0.05) and MMP-9 activity was lower in S + aVEGF than in V and S (both *p <* 0.01). Levels of MMP-2 and NLRP3 were not significantly different between groups. Nutritional supplements either alone or combined with anti-VEGF may mitigate CNV development and inhibit retinal disease involving VEGF overexpression and CNV.

## 1. Introduction

Choroidal neovascularization (CNV) is common to several retinal diseases, including pathologic myopia and age-related macular degeneration (AMD) [1,2]. Age-related macular degeneration is currently the leading cause of visual impairment and blindness in elderly patients [3] and its incidence is expected to rise as people live longer [4].

The Age-Related Eye Disease Study I (AREDS-I) showed that oral vitamin C, vitamin E, beta carotene, and zinc nutritional supplements reduce the five-year risk for developing late AMD in at-risk patients by approximately 25% [5]. However, even with AREDS-I nutritional supplements, a significant number of patients with early AMD develop CNV [6].

Proangiogenic factors have been shown to be involved in the development of CNV, with vascular endothelial growth factor (VEGF) playing the most important role [2]. Therefore, therapies that inhibit VEGF have become the gold standard for treating CNV, particularly CNV associated with AMD [7]. The main class of anti-VEGF agents block VEGF protein action and include approved (ranibizumab and aflibercept) and off-label (bevacizumab) drugs. These molecules are derived from the humanized anti-VEGF antibody, which has the ability to block VEGF and reduce retinal edema in eyes with AMD and diabetic retinopathy [8]. Blocking VEGF can stop pathological angiogenesis and reduce vessel leakage, but it does not result in regression of existing vessels [9]. Additionally, these drugs prevent blood and fluid from leaking out of abnormal vessels, but they do not address the underlying pathology that drives the disease. As a result, eyes treated with anti-VEGF agents tend to have recurrent exudation and many patients require monthly intravitreal anti-VEGF agent injections. Unfortunately, this rigorous treatment schedule can result in patient compliance issues. Moreover, VEGF is an essential factor for cell survival and sustained blockage of VEGF can lead to undesirable adverse effects [10]. Additionally, anti-VEGF agents can be very expensive. Therefore, new preventive strategies are needed to reduce the number of patients that convert from early, non-neovascular (dry) AMD to advanced, neovascular (wet) AMD.

Observational studies suggest that a higher dietary intake of lutein/zeaxanthin and/or omega-3 (ω-3) long-chain polyunsaturated fatty acids (docosahexaenoic acid (DHA) and eicosapentaenoic acid (EPA)) is associated with a decreased risk of developing advanced AMD [11,12,13,14]. Results of the AREDS II study also suggest that lutein/zeaxanthin may be more appropriate than beta carotene for AREDS-type supplements [15]. In addition, other micronutrients have been shown to have anti-angiogenic effects in both *in vitro* and *in vivo* studies [16,17]. Among these, resveratrol (3,4,5’-trihydroxy-trans-stilbene, C_14_H_12_O_3_), a commonly used nutritional supplement in Europe, has both antioxidant and anti-angiogenic effects [18,19,20,21,22,23,24]. The natural polyphenol is primarily found in grapes, red wine, and a variety of plants [18,19,20,21,22,23,24].

Here, we examine the effect of a modified AREDS II nutritional supplement, containing resveratrol, ω-3 fatty acids, glutathione, lutein, zeaxanthin, copper, zinc, selenium, and vitamins C and E, on CNV severity in a murine model of CNV. The effects of the nutritional supplement alone and in combination with anti-VEGF treatment were examined and compared to the effects of anti-VEGF monotherapy.

## 2. Materials and Methods

### 2.1. Study Animals

This study was conducted in accordance with the Association for Research in Vision and Ophthalmology (ARVO) recommendations for the use of animals in ophthalmic and vision research. The research protocol was reviewed and approved by the University of Navarra Committee on the Ethics of Animal Experiments (protocol approval number 156-11). All surgical procedures were performed under general anesthesia to minimize animal suffering.

A total of 60 C57BL6/J mice were evenly divided into four study groups (15 animals in each group). Each group was administered oral study supplements (vehicle or nutritional supplement (Resvega, Laboratoires THEA, Clermont-Ferrand, France)) and an intravitreal injection (saline or anti-VEGF agent). Both oral and intravitreal interventions are described in detail below. Mice in group V received an oral vehicle (no nutrients) and an intravitreal saline injection. Mice in group S received an oral nutritional supplement and an intravitreal saline injection. Mice in group V + aVEGF received an oral vehicle and an intravitreal anti-VEGF agent injection. Mice in group S + aVEGF received an oral nutritional supplement and an intravitreal anti-VEGF agent injection.

### 2.2. Diode Laser-Induced CNV Model

Mice were anesthetized with a mixture of ketamine (75 mg/kg, Imalgene Merial, Lyon, France,) and xylazine (10 mg/kg Rompun 2%, Bayer Animal Health, Leverkusen, Germany). Pupils were dilated with 1% tropicamide (Alcon Cusi Laboratory, Barcelona, Spain) and CNV lesions were created by using an 810 nm diode laser (Oculight SLx, Iridex, Chesham Buckinghamshire, UK). Laser settings included a relative potency scale of 250 mW, an exposure time of 0.05 s, and a spot size of 75 µm, as previously described [25]. Formation of a bubble confirmed rupturing of Bruch’s membrane (BM). Eyes that had subretinal bleeding at the time of laser application, which occurred in <10% of each group, were excluded from analyses. Eyes that were assigned to undergo molecular analyses received 12 laser applications and eyes that were assigned to undergo flat mount analyses (used to examine CNV area) received 4 laser applications [7].

Laser photocoagulation sites that developed CNV were examined 48 h and 4 weeks after laser application. Differences between treated eyes and control eyes were quantified in each study group.

### 2.3. Oral Nutritional Supplements and the Vehicle

All oral treatments (either a nutritional supplement or the vehicle) were administered once a day for 38 days by oral gavage (100 μL). Oral treatments were initiated 10 days before laser application and continued until 4 weeks (28 days) after laser application (Supplementary Material Figure S1). The vehicle was made of a mixture of water, soybean oil (Sigma Aldrich, S.A., Tres Cantos, Madrid, Spain), and Tween 80 (Sigma Aldrich) at a ratio of 50:50:1. The nutritional supplement contained resveratrol, ω-3 fatty acids, lutein, zeaxanthin, zinc, vitamin C, vitamin E, and copper, as detailed in Table 1. The amount of supplement given was determined by scaling down the manufacturer’s recommended human dose (2 capsules per day) to each animal’s body mass. The supplement was dissolved in the gavage vehicle at a ratio of 1:10 for animal administration.

**Figure 1:**
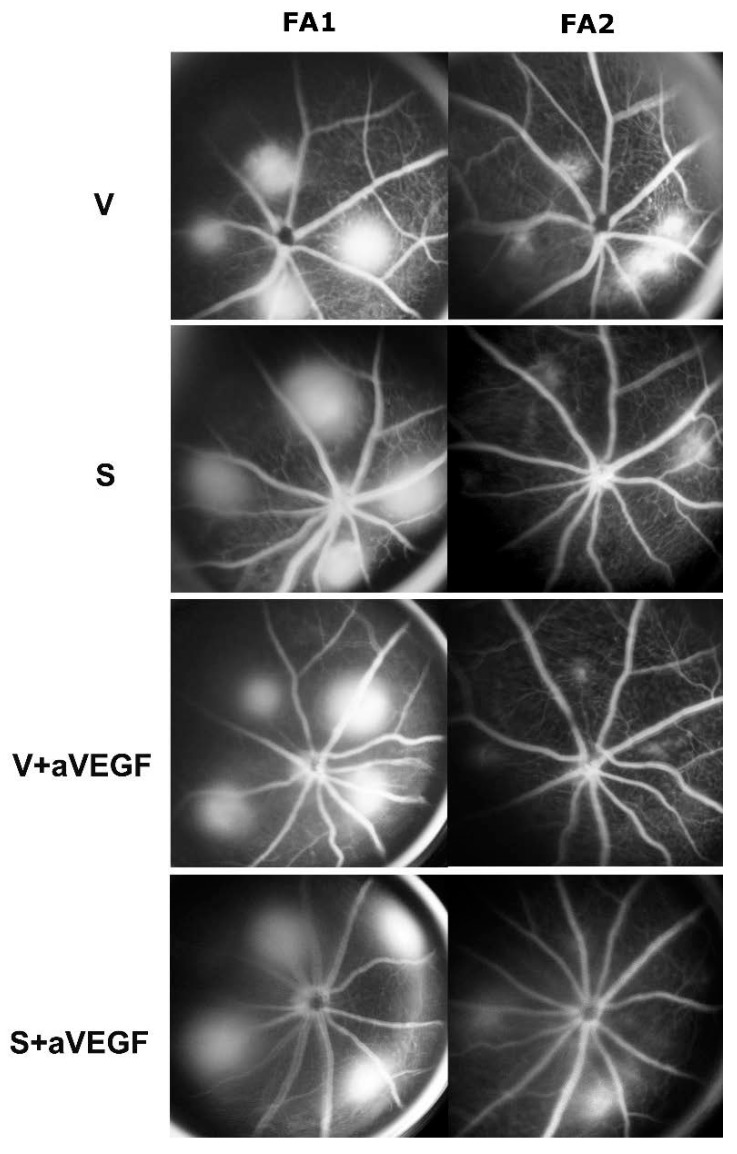
Retinal fluorescein angiography (FA). Characteristic retinal FA images obtained 48 h after laser application (FA1) and after four weeks of oral treatment (FA2) in the vehicle group (V), the nutritional supplement group (S), the vehicle and anti-vascular endothelial growth factor (VEGF) group (V + aVEGF), and the nutritional supplement + anti-VEGF group (S + aVEGF). There were 15 mice in each group. Differences between treated and vehicle groups were statistically significant (*p* < 0.05).

**Table 1:** Nutritional daily supplement composition per capsule and per mouse (assuming a mean weight of 30 g). Data are expressed in mg/kg body weight.

	Per Capsule	Murine Dose Per Day
Vitamin C (mg)	120	1.57
Vitamin E (mg)	15	0.20
Zinc (mg)	7.5	0.10
Copper (µg)	500	6.54
EPA (mg)	190	2.48
DHA (mg)	95	1.24
Lutein (mg)	5	0.07
Zeaxanthin (mg)	1	0.01
Resveratrol (mg)	15	0.20

### 2.4. Intravitreal Treatments and the Injection Procedure

Mice in the V + aVEGF and S + aVEGF groups underwent intravitreal injection of an anti-VEGF agent (1 mg/mL in 2 µL) specific to mice (LEAF™ Purified anti-mouse VEGF-A, 2G11-2A05, BioLegend, San Diego, CA, USA) 48 h after laser application. This anti-VEGF has been shown to neutralize VEGF activity in several murine models of angiogenesis [26,27]. A 30-gauge needle connected to a Hamilton syringe (Gastight 1702LT Hamilton Co., Reno, NV, USA) was used to perform the injection. Animals in the V and S groups (anti-VEGF controls) also underwent intravitreal injection, but with 2 µL of saline instead of the anti-VEGF agent.

### 2.5. Evaluation of Leakage from Choroidal Neovascular Lesions

Fluorescein angiography (FA) was performed on anesthetized mice and images were obtained using a digital camera (Topcon TRC 50FX camera, Topcon Corporation, Tokyo, Japan). Sodium fluorescein 2% (200 µL/kg) was administered intraperitoneally before images were captured. All animals underwent FA 48 h (FA1) and 4 weeks (last day of oral treatment, FA2) after laser application to assess *in vivo* CNV changes. The CNV area (in pixels) was measured using ImageQuantTL^®^ software by two independent, trained, and experienced observers who were masked to group assignments. Mean CNV area was calculated for each study group, as previously described [28], and differences between treated and control animals were assessed. This analytical and systematic approach was also used to determine whether or not changes in active CNV leakage occurred. A CNV lesion was considered inactive if lesion borders were stained by fluorescein, but no dye leakage was apparent. A CNV lesion was considered active if lesion borders were stained by fluorescein and dye leakage was present.

The following formulas were used on active CNV lesions to quantify the change in area between FA1 and FA2:

Change in Area_active CNV_ = Ratio FA2-Ratio FA1

where,

Ratio FA1 = Area_active CNV-FA1_/(Area_active CNV-FA1_ + Area_inactive CNV-FA1_)

Ratio FA2 = Area_active CNV-FA2_/(Area_active CNV-FA2_ + Area_inactive CNV-FA2_)

### 2.6. CD31 Immunofluorescence

Immunofluorescence for an endothelial cell marker (CD31) was performed on flattened retinal pigment epithelium (RPE) -choroid complexes 4 weeks after laser exposure to better assess CNV lesion size. Mice were sacrificed by CO_2_ inhalation and eyes were harvested for tissue processing. Four radial incisions were made in the sclerochoroidal “cup” to prepare choroidal flat-mounts. Tissues were incubated in a 2%–4% paraformaldehyde solution (Panreac, Castellar del Vallés, Barcelona, Spain). The RPE-choroid-sclera complex was blocked with 5% donkey serum (DakoCytomation Denmark, Glostrup, Denmark) for 30 min and bathed in rat anti-mouse CD31 (1:50, DIA-310, Dianova, Hamburg, Germany) overnight at 4 °C. A donkey anti-rat antibody labelled with fluorescein isothiocyanate (FITC, 1:200, AbD Serotec, Puchheim, Germany) was then used to label CNV lesions. Tissue samples were observed with fluorescence microscopy (Axio Imager M1, Carl Zeiss, Germany), photographed, and analyzed by three independent, masked investigators. Image J software was used to measure the size of hyperfluorescent areas, which corresponded to CNV lesions.

### 2.7. Determination of Total Protein Levels

Immediately after sacrifice, eyes were enucleated and transferred to a saline solution with a pH of 7.4 (Sigma Aldrich). Retinas were rapidly dissected by making a small incision with a scalpel blade at 1 mm behind the limbus. The incision was then extended 360° around the globe using fine ophthalmic scissors. Anterior segment structures (cornea, iris, and lens) were removed and the retina was separated from the RPE-choroidal complex. Retinas were homogenized using an Ultra-Turrax (Ika Werke GmbH & Co. KG, Staufen, Germany) to prepare them for posterior reverse transcription polymerase chain reaction (RT-PCR) analyses. The RPE-choroid samples were also homogenized, but with a Teflon pestle (Thomas Scientific, Swedesboro, NJ, USA) in 75 µL of phosphate buffer (Sigma Aldrich). The RPE-choroid samples were then centrifuged (13,000 rpm) for 20 min at 4 °C. The resulting supernatant was collected and protein concentration was determined using a slightly modified Bradford assay (Bio-Rad, Hercules, CA, USA) [28].

### 2.8. Vascular Endothelial Growth Factor and NOD-like Receptor Family Pyrin Domain Containing 3

The VEGF and NOD-like receptor family pyrin domain containing 3 (NLRP3) proteins were quantified from RPE homogenates. Equal amounts of RPE homogenate (3.5 µg) were mixed with Laemmli buffer (4× NuPage, Invitrogene) and boiled for 5 min. Samples were separated in 12% sodium dodecyl sulfate polyacrylamide gel electrophoresis (SDS-PAGE) gels and transferred to nitrocellulose membranes (GE Healthcare). After blocking with a 2% w/v Advance™ blocking agent (GE Healthcare, Fairfield, CT, USA), and incubating with a 0.1% w/v Tween^®^-20 in tris-buffered saline (TBS) for 1 h at room temperature, membranes were exposed to an anti-VEGF antibody (1:1000, sc7269, Santa Cruz Biotechnology Inc., Santa Cruz, CA, USA) overnight at 4 °C or an anti-NLRP3 antibody (1:1000, Epitomics, Abcam, Madrid, Spain) for 2 h at room temperature. Membranes were then incubated with a horseradish peroxidase conjugated goat anti-mouse antibody (sc2005; 1:5000, Santa Cruz Biotechnology Inc.) or a goat anti-rabbit antibody (sc2054; 1:5000 Santa Cruz Biotechnology Inc.). Chemoluminescent signals were detected with an enhanced chemoluminescence (ECL) kit (ECL-Advance™ Western Blotting Detection Kit, GE Healthcare) and signals were measured using an ImageQuant 400 (GE Healthcare). The relative intensities of immunoreactive bands were quantified with ImageQuant TL software (GE Healthcare). Loading was verified by Ponceau S (Sigma Aldrich) red staining solution and VEGF levels were normalized with the same blot by stripping and reblotting with an anti-β-actin monoclonal antibody (1:10,000; Sigma Aldrich).

### 2.9. Quantitative Real Time-Polymerase Chain Reaction

Total RNA was isolated from mice tissue using an ABI PRISM™ 6100 Nucleic Acid PrepStation (Life Technologies, Carlsbad, CA, USA). Subsequently, the quantity and quality of purified messenger RNA (mRNA) was checked using a NanoDrop spectrophotometer (Nanodrop Technologies, Montchanin, DE, USA) at 260/280. Using the qScript cDNA Supermix Kit (Quanta Biosciences, Inc., Gaithersburg, MD, USA), 1000 ng of each mRNA was reverse transcribed under the following conditions: 5 min at 25 °C, 30 min at 42 °C, and 5 min at 85 °C using a 2720 Thermal Cycler (Life Technologies). Three pre-designed and validated gene-specific TaqMan Gene Expression Assays (*VEGFa*- Mm 01281449_m1, *MMP2*- Mm 00439498_m1, *and MMP9*- Mm 00442991_m1; Applied Biosystems, Life Technologies, Carlsbad, California, USA) were used to conduct RT-PCR. The PCR reaction volume was 20 µL containing 1 µL cDNA, 10 µL TaqMan 2× Universal PCR Master Mix (Life Technologies), 1 µL validated gene-specific TaqMan Gene Expression Assay 20× (Life Technologies), and 8 µL water. An ABI Prism 7300 Real-Time PCR System (Life Technologies) was used for amplification with the following protocol: 2 min at 50 °C, 10 min at 95 °C, 42 cycles of 15 s at 95 °C, and 1 min annealing and extension at 60 °C. Two housekeeping genes (*glyceraldehyde 3-phosphate dehydrogenase* (*GAPDH*) 4352932E and *beta-actin*) were used as internal controls. However, only *GAPDH* was used for statistical evaluations because it was the most reliable of our samples. Relative quantification studies were performed on collected data using 7300 System SDS software 1.3 (Life Technologies). The relative quantity (RQ) of the gene-specific mRNA was calculated using DataAssist™ 2.0 (Life Technologies).

### 2.10. Matrix Metalloproteinase-2 and -9 Activity

The activities of MMP-2 and MMP-9 in RPE-choroid homogenates were quantified using gelatin zymography. An 8-μg sample of homogenate supernatant total protein was mixed with a non-reducing sample buffer (62.5 mM Tris-HCl, 10% glycerol, 0.1% bromophenol blue; pH = 6.8) and directly electrophoresed in 9% SDS-PAGE containing 0.1% w/v gelatin. After electrophoresis, gels were washed 4 times in a 2.5% v/v Triton X-100 solution to remove excess SDS (20 min each at room temperature), transferred to a solution (Zymogram Development Buffer, Bio-Rad), and incubated for at least 18 h at 37 °C. Protein fixation was achieved by incubating the gels for 15 min with 50% methanol/7% acetic acid and then washing them for a total of 30 min (six 5-min washes) with distilled water. Afterwards, gels were stained for 1 h with GelCode Blue Stain Reagent (Pierce, Rockford, IL, USA), counterstained with distilled water, and analyzed with ImageQuant TL software (GE Healthcare) after densitometric scanning. The MMP activation ratio for MMP-2 was calculated as active MMP intensity/(active MMP + proMMP) intensity. The MMP-9 activity was also determined using intensity measurements. Each zymography assay was repeated at least three times to ensure accuracy.

### 2.11. Statistical Analyses

Data are reported as mean ± standard error of the mean (SEM) where applicable. Differences between study groups that were found to be significant with analysis of variance (ANOVA) or Kruskal-Wallis tests were also examined using a *post hoc* Bonferroni’s correction or Mann-Whitney U test for multiple comparisons. Statistical significance was defined as *p* < 0.05 and analyses were performed using SPSS statistical software (version 15.0, SPSS, Inc., Chicago, IL, USA).

## 3. Results

### 3.1. Effect of Nutritional Supplements on Choroidal Neovascular Lesion Leakage

**Figure 2:**
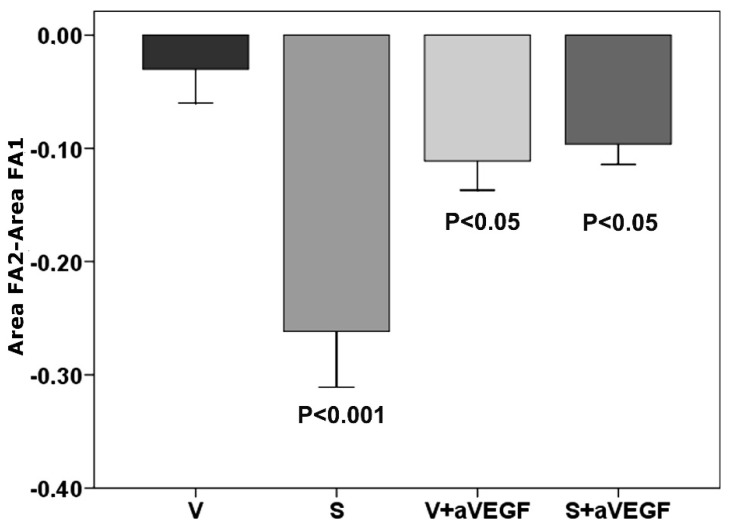
Fluorescein leakage. A decrease in active vessel area compared to the vehicle group (V) was apparent in all treated groups (nutritional supplement (S), supplement and anti-vascular endothelial growth factor agent (S + aVEGF), and vehicle and anti-VEGF agent (V + aVEGF)). There were 15 animals in each group and data are expressed as mean ± standard error of the mean.

Analyses of FA images showed a highly significant reduction in CNV lesion leakage in the nutritional supplement group (group S) compared to the vehicle group (group V, *p* < 0.001). A significant difference in CNV lesion leakage was also found between the V+aVEGF and S+aVEGF groups (*p* < 0.05; Figure 1 and Figure 2, Table 2).

**Table 2:** Summary of study results. Mean choroidal neovascularization (CNV) area, as assessed by fluorescein angiography (FA) and CD31 (measured in pixels). Vascular endothelial growth factor (VEGF) and NLRP3 protein expression levels and MMP-2 and MMP-9 activity (in percentage of arbitrary units [AU]) compared to the vehicle group (V). Data are shown as mean ± standard error of the mean. Real time polymerase chain reaction analysis results are expressed as specific gene logarithm relative quantity (Log RQ) and the p-value is provided. The control gene was β-actin.

	V	S	V + aVEGF	S + aVEGF
FA	−0.030 ± 0.003	−0.261 ± 0.04 ***	−0.112 ± 0.025 *	−0.091 ± 0.03 *
CD31 (% *vs.* V)	100 ± 10.03	63 ± 5.35 **	80 ± 9.62 *	64 ± 9.69 *
*Protein expression*				
VEGF (% *vs.* V)	100 ± 8.11	74.78 ± 11.96 *	67.39 ± 10.30 *	68.59 ± 9.70 *
NLRP3 (% *vs.* V)	100 ± 13.12	102.07 ± 4.26	108.08 ± 13.20	90.8 5 ± 12.83
*Enzymatic activity*				
MMP2 (% *vs.* V)	100 ± 6.49	80.45 ± 9.93	83.25 ± 6.96	104.30 ± 11.41
MMP9 (% *vs.* V)	100 ± 10.08	105.50 ± 12.81	73.79 ± 10.64	54.75 ± 5.32 **, **^†^**
*Gene expression (Log RQ)*				
VEGF	0.00	−0.53 *	−0.48 *	−0.54 *
MMP2	0.00	−0.25	−0.11	−0.70
MMP9	0.00	−0.18	−0.09	−0.29 ***

S = dietary supplement group, V + aVEGF = vehicle plus intravitreal VEGF group; S + aVEGF = dietary supplement plus intravitreal VEGF group. * indicates *p* < 0.05 *vs.* V; ** indicates *p* < 0.01 *vs.* V; *** indicates *p* < 0.001 *vs.* V; **^†^** indicates *p* < 0.05 *vs.* S.

### 3.2. Effect of Nutritional Supplements on Choroidal Neovascular Lesion Size

**Figure 3:**
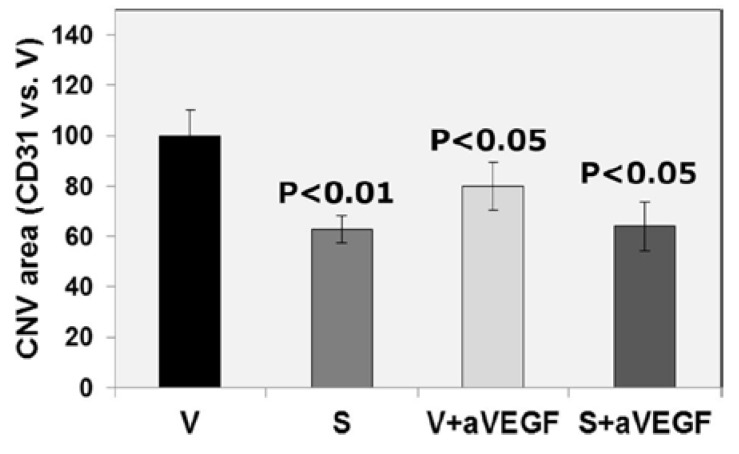
Area of choroidal neovascularization (CNV), as measured with CD31 immunofluorescence. The area of CNV was smaller in all treated groups (nutritional supplement (S, *p <* 0.01), supplement and anti-vascular endothelial growth factor agent (S + aVEGF, *p <* 0.05), and vehicle and anti-VEGF agent (V + aVEGF, *p <* 0.05)) than in the vehicle (V) group. There were 15 animals in each group and data are expressed as mean ± standard error of the mean.

**Figure 4:**
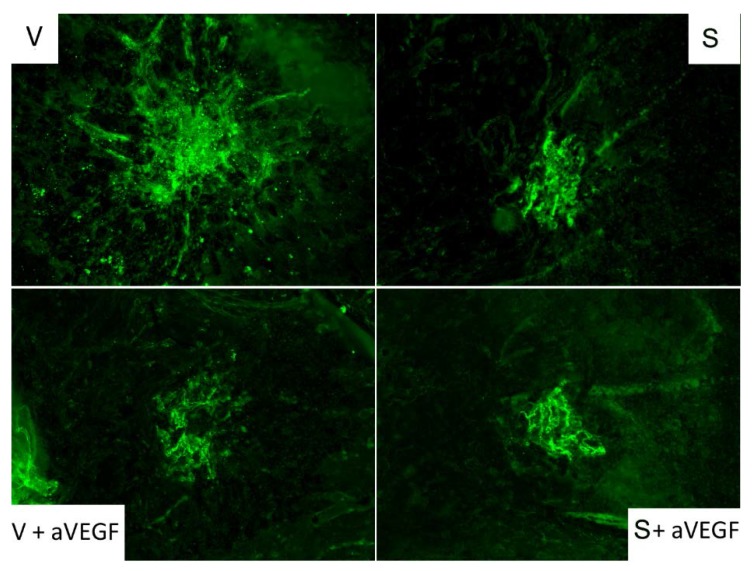
Choroidal neovascularization (CNV) detection by CD31 immunofluorescence. Mice that received only the vehicle (upper left, group V) had larger areas of CNV than mice that received the supplement (upper right) and/or anti-vascular endothelial growth factor (VEGF) treatment. Treatment groups included the nutritional supplement (S) group, the supplement and anti-vascular endothelial growth factor agent (S + aVEGF) group, and the vehicle and anti-VEGF agent (V + aVEGF) group.

The size of CNV lesions, measured with CD31 immunofluorescence, was smaller in group S than in group V (*p* < 0.01). Lesion size was also smaller in mice receiving anti-VEGF therapy (groups S + aVEGF and V + aVEGF) than in group V mice (*p* < 0.05). Interestingly, there were no significant differences in lesion size between mice in group S and mice treated with the anti-VEGF agent alone or combined with supplement (Table 2, Figure 3 and Figure 4).

### 3.3. Effect of the Nutritional Supplement on Vascular Endothelial Growth Factor and NOD-Like Receptor Family Pyrin Domain Containing 3

**Figure 5:**
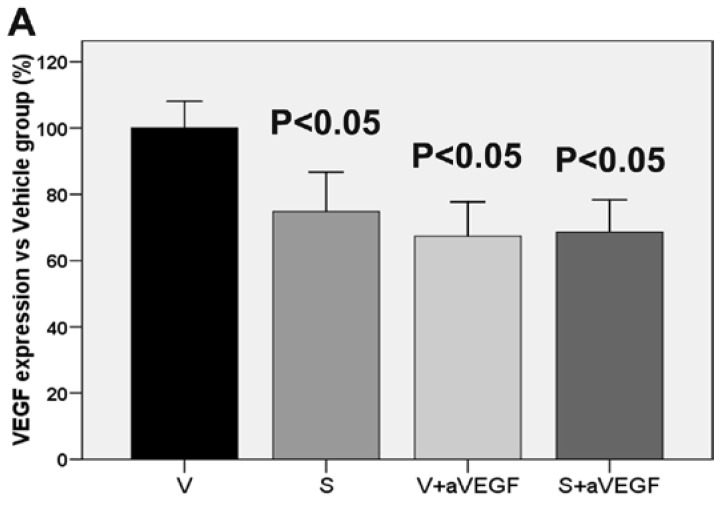

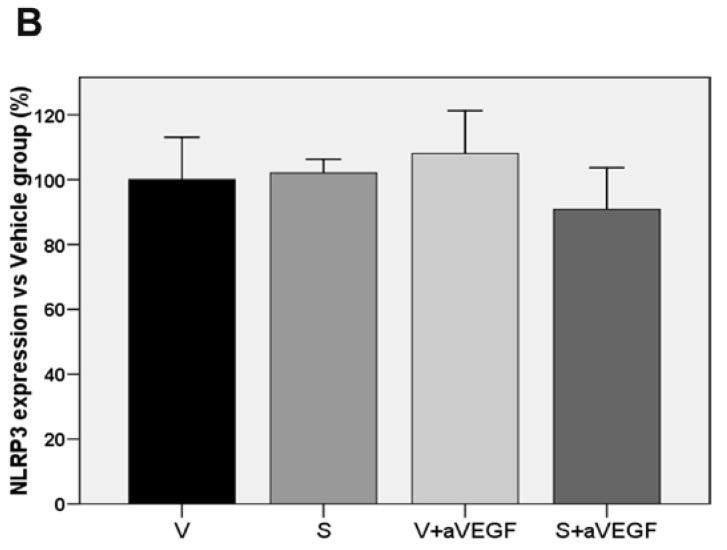
Vascular endothelial growth factor (VEGF) and NLRP3 protein expression in retinal tissue. A: Representative VEGF expression in all experimental groups (*n* = 10, β-actin was blotted as an internal control. Differences between all treated groups (nutritional supplement [S], supplement and anti-vascular endothelial growth factor agent (S + aVEGF), and vehicle and anti-VEGF agent (V + aVEGF)) and the vehicle (V) group were statistically significant (*p* < 0.05). B: Expression of NLRP3 protein in retinal tissue. No significant differences were found between study groups.

Expression of the VEGF protein was lower in all treated groups (groups S, V + aVEGF, and S + aVEGF) than in group V (all *p* < 0.05). Additionally, VEGF expression was not significantly different between the S and S + aVEGF groups (Table 2, Figure 5). Expression of the NLRP3 protein was not significantly different between any of the four study groups (Figure 5).

### 3.4. Matrix Metalloproteinase Activity

**Figure 6:**
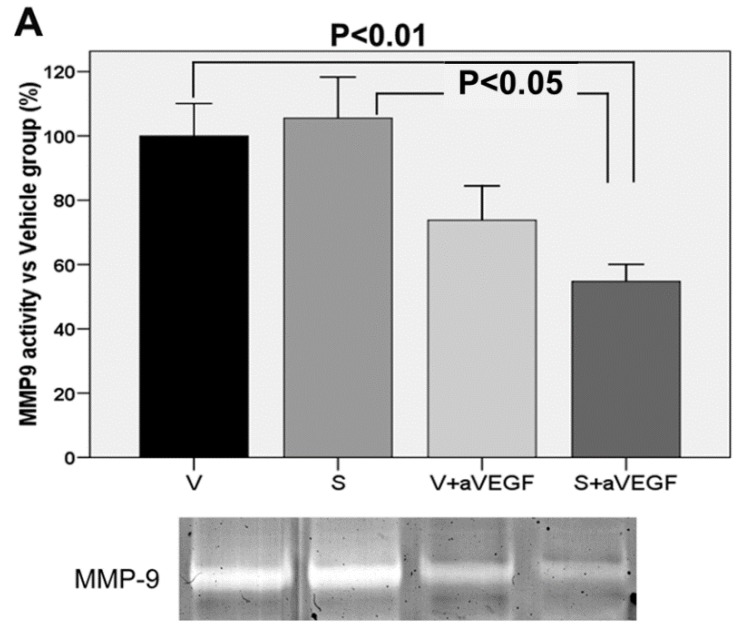

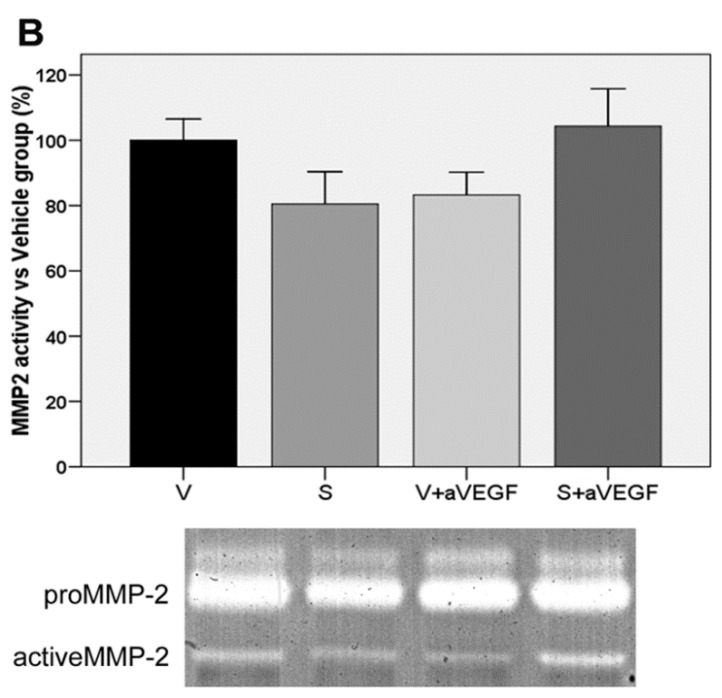
Enzymatic activity of matrix metalloproteinase (MMP)-9 (**A**) and MMP-2 (**B**). A: Upper: Representative MMP9 expression graphs (mean ± standard error of the mean (SEM)) for all experimental groups (*n* = 10 eyes, β-actin was blotted as an internal control). Treatment groups included the nutritional supplement (S), the supplement and anti-vascular endothelial growth factor agent (S + aVEGF), and the vehicle and anti-VEGF agent (V + aVEGF) groups. Differences between groups S + aVEGF and V and between groups S and S + aVEGF were statistically significant (both *p* < 0.01). Lower: Representative MMP-9 activity zymograms for all study groups. B: Upper: Representative MMP-2 expression graph (mean ± SEM) for all experimental groups (*n* = 10 eyes). No significant differences between groups were observed. Lower: Representative MMP-2 activity zymograms for all study groups.

Activity of the MMP-9 protein was lower in group S + aVEGF than in group V (*p* < 0.01). It was also lower in group S + aVEGF than in group S (*p* < 0.05). However, there was no significant difference in MMP-9 activity between groups V and S (Table 2, Figure 6). Expression of the MMP-2 protein was not significantly different between any of the four study groups (Table 2, Figure 6).

### 3.5. Effect of the Nutritional Supplement on Gene Expression

Gene expression of VEGF, MMP-2, and MMP-9 in each of the four study groups is summarized in Table 2. Expression of the VEGF gene was higher in group V than in group S (*p* < 0.05), group V + aVEGF (*p* < 0.05), and group S + aVEGF (*p* < 0.05). Additionally, MMP-9 gene expression in group V was significantly higher than that of group S + aVEGF (*p* < 0.001), but not that of groups S and V + aVEGF. Expression of the MMP-2 gene was not significantly different between any of the four study groups.

## 4. Discussion

To the best of our knowledge, this is the first study to investigate the efficacy of a modified AREDS II, resveratrol-containing vitamin and mineral nutritional supplement in reducing early laser-induced CNV in mice. The main findings of this study were the reductions in CNV leakage and lesion size, compared to controls, in mice given an oral nutritional supplement in combination with an intravitreal anti-VEGF monoclonal antibody. The CNV leakage and lesion size were also reduced in mice receiving only the intravitreal anti-VEGF antibody or only the nutritional supplement. In animals treated with the nutritional supplement, these effects were also accompanied by a decrease in VEGF and MMP-9 gene expression and protein activity levels. However, differences between an animal model of CNV and human wet AMD are obvious, and although interesting, our results do not support nutritional supplement monotherapy for CNV in humans. Recent studies have shown vision improvements in patients with AMD-associated CNV after nutritional supplement intake [29,30], but no other clinical evidence supports antioxidant supplement monotherapy for treating wet AMD.

The prevalence of AMD is dramatically increasing as the proportion of elderly people in the Western world continues to rise [31]. No definitive treatment is available for AMD, but vitamin and mineral supplements used for secondary prevention may reduce the incidence of advanced AMD. Based on AREDS-I, AREDS-II, and observational food studies, clinicians currently recommend that eligible AMD patients take nutritional supplements and consume foods rich in antioxidants, both of which impact disease management [32]. In a recent study at an American tertiary retinal center, 93% of patients were taking dietary supplements [33], but some patients still developed wet AMD. Recombinant antibodies that bind to and inhibit VEGF have been adapted for intravitreal application in patients with wet AMD [34]. Patients with wet AMD in one eye are at a greater risk of also developing CNV in the other eye. These patients are generally treated with intravitreal anti-VEGF drugs in the affected eye and with nutritional supplements for the unaffected eye.

Despite the widespread use of nutritional supplements for managing AMD, few studies have examined the effect of these supplements on CNV [29,30]. Many studies have examined the effect of single ingredient dietary supplements, but such isolated constituents are neither used nor recommended for mitigating advanced AMD [5,6]. Multiple nutrients are thought to decrease oxidative stress, which is thought to play a central role in AMD pathogenesis [2]. Previous research by our group found that a multivitamin-mineral complex with vitamins C and E and flavonoids reduced oxidative stress and ultrastructural retinal changes in a murine model of hypercholesterolemia [35]. We have also found that zeaxanthin and multivitamin supplements may delay or reverse RPE changes and BM deposits and reduce VEGF expression in apoE^−/−^ mice [36]. In addition, ω-3 fatty acids have been shown to have an antiangiogenic effect in CNV models and an anti-proliferative effect on endothelial cells [37,38,39,40]. The nutritional supplement used in this study also contained ω-3 fatty acids, which may have contributed to its potential antiangiogenic effect. Evidence suggests that resveratrol also has antiangiogenic properties, as demonstrated in a mouse model of macular telangiectasia (fewer CNV lesions in resveratrol-treated mice than in controls) [41].

It has previously been shown that high doses of resveratrol (subcutaneous or intraperitoneal) can inhibit laser-induced CNV [42,43]. Because this study was designed to mimic clinical practice, we examined the effect of a relatively low dose of orally-delivered resveratrol, given with other common micronutrients, on CNV lesions.

The effect of oral resveratrol (25–50 mg/kg/day) has been shown to reduce macrophage infiltration and inflammatory and angiogenic (VEGF) cytokines in the RPE-choroid complex in a murine model of laser CNV [44]. Kanavi *et al.* [45] found that resveratrol inhibited Akt/protein kinase B activity in choroidal endothelial cells, which is consistent with the anti-migratory properties of resveratrol that have been observed *in vitro*. Resveratrol also inhibits branching and network formation of choroidal endothelial cells, *in vitro* correlates of angiogenesis. Our results are consistent with these observations. However, our main objective was to examine the effect of the complete dietary supplement and not to test the effect of resveratrol alone. Additionally, the resveratrol dose used in the current study (20 mg/kg/day) was lower than that administered by others for resveratrol monotherapy. However, when this lower dose of resveratrol was combined with different micronutrients, its antiangiogenic effects may have been enhanced. Furthermore, the duration of the treatment was longer in our study (38 days) compared to previous studies (five days), suggesting that longer treatment may have larger effects than shorter treatment. Lastly, both this study and previous studies suggest that lower resveratrol doses may reduce CNV when coupled with other bioactive agents. Previous studies on the molecular mechanism underlying CNV development have reported that VEGF is a critical angiogenic factor [46]. We hypothesize that our observed reduction in VEGF protein and gene expression resulted in a reduction in CNV leakage. Our previous studies showed that oral lutein, zeaxanthin, and multivitamins reduce retinal VEGF expression [36,47]. Other nutrients in our supplement may have also decreased VEGF expression [41,48,49,50].

In our study, reductions in VEGF protein levels and VEGF gene expression were observed in both groups treated with the nutritional supplement, but differences between these two groups were not significant. Therefore, the effects of the dietary supplement and anti-VEGF therapy were not likely synergistic. We hypothesize that the protective effect of the nutritional supplement was masked by the therapeutic effect of the anti-VEGF treatment. Therefore, our results are consistent with the idea that, in this mouse model, our nutritional supplement reduced VEGF-related vascular pathology in early CNV lesions.

In agreement with our results, Rezende *et al.* [51] recently showed that ω-3 fatty acid supplementation in combination with anti-VEGF therapy is associated with decreased vitreous VEGF levels in AMD patients with CNV. These authors hypothesized that factors other than VEGF-A may contribute to CNV activity in eyes with wet AMD. As a result, they suggested that combination therapy with other agents is likely necessary for many patients to completely halt disease activity and promote CNV regression.

Extracellular remodelling by MMPs may also be involved in pathological angiogenesis in laser-induced CNV. These enzymes are constitutively expressed, but under certain conditions, including pathological angiogenesis, their expression can increase. In support of this, MMP-9 accumulates in the BM of eyes with AMD [52].

In the current study, MMP-9 gene expression and protein activity were lower in the group treated with both the supplement and an anti-VEGF antibody (group S + aVEGF) than in the group treated with the vehicle (group V). These findings suggest that VEGF and MMPs affect each other and that VEGF inhibitors may also affect MMP production [53]. Therefore, nutritional supplements and anti-VEGF agents likely have synergistic effects when administered in combination. Previous studies showed that resveratrol, ω-3 fatty acids, and anti-VEGF agents are all capable of reducing MMP-9 levels [18,49,50,53,54,55,56,57] with possibly synergistic effects. Although we did not find significant differences in MMP-9 activity in mice treated with anti-VEGF antibody or nutritional supplement monotherapy, we unexpectedly found that the combination of both treatments significantly reduced MMP-9 activity by 45% compared to other groups.

Because both VEGF gene and protein expression were reduced in the dietary supplemented groups (S and S + aVEGF groups), we suggest that the dietary supplement suppresses VEGF. The only parameter that was synergistically affected by the dietary supplement and an intravitreal anti-VEGF agent was MMP-9 gene expression and activity. Matrix remodelling is an essential process for CNV pathogenesis and subsequent fibrosis. We recently reported a decrease in MMP-9 gene expression in a previous study on a rat CNV model treated with anti-TGFβ peptides [58]. The results of the current study confirm that MMP-9 gene expression and activity is changed by the supplement. Furthermore, the supplement likely functions by reducing VEGF and by acting on other related pathways, including the TGFβ signalling pathway.

The role of MMP-2 in AMD is somewhat controversial. Both resveratrol and ω-3 fatty acids can independently suppress MMP-2 protein activity and RNA expression in patients with multiple myeloma [54]. However, other studies did not show any inhibition of MMP-2 [57,59]. In the current study, which examined resveratrol, ω-3 fatty acids, and other micronutrients in combination, MMP-2 levels were not significantly different between study groups. Therefore, even though MMP-9 can regulate VEGF bioactivity towards pathological angiogenesis, the regulatory effects of MMP-2 are different [60,61].

Inflammation is believed to play an important role in the development of AMD [59,60]. Therefore, we investigated the effect of our nutritional supplement on inflammasome-related proteins. The NLRP3 protein may be involved in CNV development because it is expressed in response to tissue injury, directly protecting the eye against AMD [62]. However, NLRP3 expression has been associated with both geographic atrophy and neovascular AMD because it was detected in drusen and near the BM [63]. Additionally, laser-induced CNV lesions were recently observed to be bigger in NLRP3^−/−^ mice than in wild type animals [63]. Taken together, these studies suggest that the NLRP3 inflammasome has distinct roles in both wet and advanced dry AMD and that it may influence which form of the disease develops. In our study, a decrease in CNV leakage (as observed on FA) occurred without a corresponding decrease in NLRP3 expression. Therefore, this pathway may be more related to CNV in AMD subtypes with a low degree of chronic RPE inflammation [62,63]. Therefore, our model of CNV represents acute laser rupture of the BM, a condition that is likely less influenced by inflammatory processes than AMD in humans.

Our study had several limitations. Given the differences between human and mouse retinas, our results cannot be easily transferred to the clinical situation. Rodents with CNV may have higher reactions to substances than humans with CNV. Neovascular AMD and experimental CNV may be related, but we cannot say how strong this relationship is. The early physiological development of CNV lesions in our mouse model may have been reduced or slowed as a result of natural, intrinsic, antiangiogenic growth factor release. It should also be noted that we induced CNV by producing iatrogenic lesions with a laser in young, healthy mice with a healthy RPE. Our study was also limited by the number of study groups and time points chosen. Examining ω-3 fatty acids and resveratrol separately would have been helpful, as would have examining interim time points before 28 days. However, because we did observe statistically significant differences at 28 days, it is logical to assume that similar differences would have been present between 14 and 21 days, when pathological angiogenesis may have been even more active. If we had not found these differences at 28 days, it would have made sense to examine earlier time points to see if the relevant time interval had been missed.

## 5. Conclusions 

In summary, administration of a nutritional supplement, either alone or in combination with an anti-VEGF antibody, appeared to decrease early CNV progression in mice. Reductions in CNV lesion size and fluorescein leakage were associated with VEGF and MMP-9 level reductions. These findings indicate that VEGF affects CNV by a similar mechanism as several micronutrients (e.g., resveratrol and ω-3 fatty acids) that have beneficial effects on CNV. Additionally, the nutritional supplement, alone or in combination with anti-VEGF therapy, may help mitigate CNV development in mice with laser-induced lesions. Therefore, these nutrients may be useful in preventing retinal disease related to VEGF overexpression and CNV. Our results support the need for further longitudinal research in humans to validate the efficacy of nutritional supplements in patients at risk for developing wet AMD.

## Supplementary Files

Supplementary File 1
