# Supplementary material for: Modifying Choroidal Neovascularization Development with a Nutritional Supplement in Mice
Source: Nutrients. 2015 Jul 6;7(7):5423–42. doi: 10.3390/nu7075229 (PMC4517006; doi:10.3390/nu7075229)
Supplement: Supplementary File 1 [file nutrients-07-05229-s001.docx]

Supplementary Materials


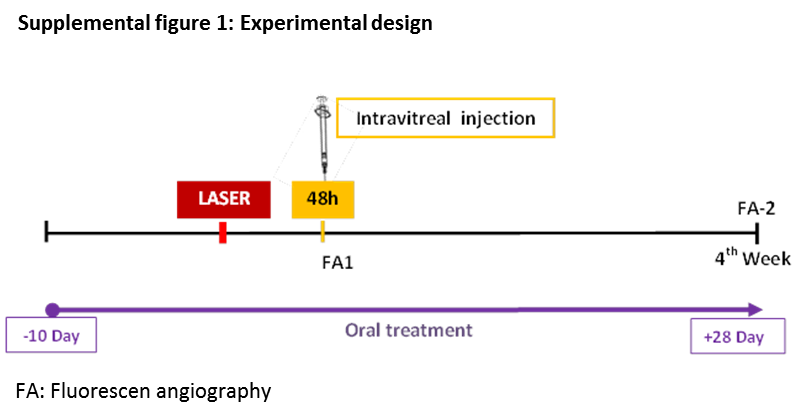


**Figure S1.** Experimental design. Schema of the experimental design and the time of nutritional supplement administration.

© 2015 by the authors; licensee MDPI, Basel, Switzerland. This article is an open access article distributed under the terms and conditions of the Creative Commons Attribution license (http://creativecommons.org/licenses/by/4.0/).
